# Lanthanide-Dependent Methanol and Formaldehyde Oxidation in *Methylobacterium aquaticum* Strain 22A

**DOI:** 10.3390/microorganisms8060822

**Published:** 2020-05-30

**Authors:** Patcha Yanpirat, Yukari Nakatsuji, Shota Hiraga, Yoshiko Fujitani, Terumi Izumi, Sachiko Masuda, Ryoji Mitsui, Tomoyuki Nakagawa, Akio Tani

**Affiliations:** 1 Institute of Plant Science and Resources, Okayama University, Okayama 710-0046, Japan; p5vd9ik5@s.okayama-u.ac.jp (P.Y.); ag422066@s.okayama-u.ac.jp (Y.N.); padi0iqc@s.okayama-u.ac.jp (S.H.); fujitani.y@gmail.com (Y.F.); ggkwcrn200@gmail.com (T.I.); sachiko.masuda@riken.jp (S.M.); 2 Advanced Low Carbon Technology Research and Development Program, Japan Science and Technology Agency, Tokyo 102-0076, Japan; 3 RIKEN Center for Sustainable Resource Science, Kanagawa 230-0045, Japan; 4 Department of Biochemistry, Faculty of Science, Okayama University of Science, Okayama 700-8530, Japan; rmitsui@dbc.ous.ac.jp; 5 The United Graduate School of Agricultural Science, Gifu University, Gifu 501-1193, Japan; t_nakaga@gifu-u.ac.jp; 6 The Graduate School of Natural Sciences and Technologies, Gifu University, Gifu 501-1193, Japan

**Keywords:** lanthanide, methylotroph, XoxF, methanol dehydrogenase, *Methylobacterium* species

## Abstract

Lanthanides (Ln) are an essential cofactor for XoxF-type methanol dehydrogenases (MDHs) in Gram-negative methylotrophs. The Ln^3+^ dependency of XoxF has expanded knowledge and raised new questions in methylotrophy, including the differences in characteristics of XoxF-type MDHs, their regulation, and the methylotrophic metabolism including formaldehyde oxidation. In this study, we genetically identified one set of Ln^3+^- and Ca^2+^-dependent MDHs (XoxF1 and MxaFI), that are involved in methylotrophy, and an ExaF-type Ln^3+^-dependent ethanol dehydrogenase, among six MDH-like genes in *Methylobacterium aquaticum* strain 22A. We also identified the causative mutations in MxbD, a sensor kinase necessary for *mxaF* expression and *xoxF1* repression, for suppressive phenotypes in *xoxF1* mutants defective in methanol growth even in the absence of Ln^3+^. Furthermore, we examined the phenotypes of a series of formaldehyde oxidation-pathway mutants (*fae1*, *fae2*, *mch* in the tetrahydromethanopterin (H_4_MPT) pathway and *hgd* in the glutathione-dependent formaldehyde dehydrogenase (GSH) pathway). We found that MxaF produces formaldehyde to a toxic level in the absence of the formaldehyde oxidation pathways and that either XoxF1 or ExaF can oxidize formaldehyde to alleviate formaldehyde toxicity in vivo. Furthermore, the GSH pathway has a supportive role for the net formaldehyde oxidation in addition to the H_4_MPT pathway that has primary importance. Studies on methylotrophy in *Methylobacterium* species have a long history, and this study provides further insights into genetic and physiological diversity and the differences in methylotrophy within the plant-colonizing methylotrophs.

## 1. Introduction

**Figure 1:**
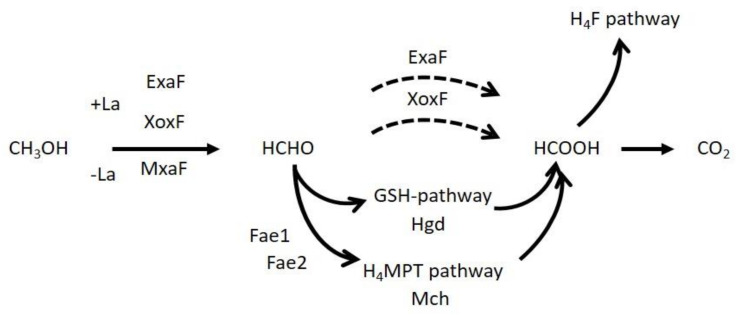
A schematic of the methylotrophy pathway. Dashed lines indicate possible direct oxidations.

Methanol is the most abundant single-carbon compound produced by plants in pectin metabolism, primarily during leaf expansion, and is emitted through the stomata [1]. It can be metabolized by methylotrophic bacteria as a sole carbon and energy source [2,3]. Methylotrophs play an important role as methanol consumers in the phyllosphere and have received significant research attention [4,5,6]. *Methylobacterium extorquens* strain AM1, recently reclassified as *Methylorubrum extorquens* strain AM1 [7] (hereafter referred to as strain AM1) has long served as a model organism of Gram-negative methylotrophs for biochemical and physiological studies of methanol metabolism [8,9]. The metabolic process of strain AM1 begins with the oxidation of methanol into formaldehyde in the periplasm using pyrroloquinoline quinone (PQQ)-dependent methanol dehydrogenases (MDHs) [2,10]. Formaldehyde is then oxidized into formate within the cytoplasm through the tetrahydromethanopterin (H_4_MPT) pathway. This pathway is catalyzed by formaldehyde-activating enzyme (Fae), methylene-H_4_MPT dehydrogenases MtdA and MtdB, methenyl-H_4_MPT cyclohydrolase (Mch), and formyltransferase/hydrolase complex (Fhc) [11,12]. Formate is then finally oxidized to CO_2_ by formate dehydrogenases (Fdh) [11,13] or converted into methylene-tetrahydrofolate via the tetrahydrofolate (H_4_F) pathway for assimilation [12,14] (Figure 1).

PQQ-dependent MDHs can be classified into two types: MxaFI and XoxF. MxaFI is a heterotetrameric (α_2_β_2_), calcium (Ca^2+^)-dependent MDH [15], whereas XoxF is a dimeric lanthanide (Ln^3+^)-dependent MDH [16,17,18,19]. The recent findings regarding the Ln^3+^ dependency of XoxF have expanded our knowledge of methylotrophy and generated new questions. First, the wider distribution of *xoxF* in many bacterial genomes led to the discovery of new methylotrophs of novel genera [20,21,22] and the discovery that certain known microorganisms are methylotrophs [23,24]. Second, bacteria containing both MDHs, including strain AM1, exhibit a phenomenon called the “lanthanide switch”. XoxF1 expression is induced in the presence of Ln^3+^, whereas MxaFI is expressed when Ln^3+^ is absent [25,26,27]. In strain AM1, the lanthanide switch is controlled by a two-component regulation system (TCRS) consisting of MxbD (sensor) and MxbM (regulator), which is responsible for the expression of *mxaF* and the repression of *xoxF1* in the absence of Ln^3+^ [27,28,29]. The ligand of MxbD is currently unknown but has been suggested to be XoxF1 [27,30], because *mxaF* expression is dependent on the presence of *xoxF1* in addition to *mxbDM* and *mxcQE* (encoding another set of TCRS). Third, a set of genes encoding the TonB-dependent receptor and the ABC transporter, which are now classified as the *lut* (lanthanide utilization and transport) genes, is essential for Ln^3+^-dependent methylotrophy in strain AM1 and also in *M. extorquens* strain PA1 [30,31]. These findings suggest that Ln^3+^ is incorporated into the cytosol; the observation of the phosphate-salt of Ln^3+^ in the cytosol supports this possibility [31]. Fourth, in addition to XoxF1, another Ln^3+^-dependent MDH homolog named ExaF participates in methylotrophy in strain AM1. ExaF oxidizes ethanol and acetaldehyde as well as methanol and formaldehyde [32]. Finally, the XoxF proteins encoded in many bacterial genomes can be categorized into five major groups (groups XoxF1–XoxF5, [33]). XoxF-type MDHs have been characterized from various species including *Methylacidiphilum infernorum*, *Paracoccus denitrificans*, *Methylotenera mobilis* [34], *Methylacidiphilum fumariolicum* SolV [18], and *Methylomirabilis oxyfera* [35].

*Methylobacterium aquaticum* strain 22A (hereafter referred to as strain 22A) was isolated from the moss *Racomitrium japonicum* [36] with which it engages in beneficial symbiotic interaction [37]. Our transcriptome (RNA-seq) analysis of strain 22A showed distinct gene regulation affected by the presence of Ln^3+^ [38]. First, among the six MDH-like genes encoded in the genome, the expression of the *mxa* cluster was downregulated and that of the *xox* cluster was upregulated; a gene (Maq22A_c07235) encoding a putative ExaF-type alcohol dehydrogenase (ADH) was also upregulated. Second, the genes for formaldehyde oxidation (H_4_MPT pathway and glutathione-dependent formaldehyde dehydrogenase, GSH pathway) were downregulated. Third, strain 22A cells grown in the presence of La^3+^ showed reduced production of formaldehyde through methanol oxidation compared to those grown in the absence of La^3+^. These observations raise important questions regarding the metabolic enzymes in the methylotrophic pathways.

Based on the recent reclassification of *Methylobacterium* species, strain AM1 and strain 22A differ at the genus level, prompting us to study the methylotrophy in strain 22A in greater depth as another model for methylotrophy. There are several differences between the two strains: while strain AM1 has two functional XoxF genes (XoxF1 and XoxF2) [27], the second XoxF gene in strain 22A seems to be a pseudogene with low transcription [38]. In addition to the H_4_MPT pathway, strain 22A contains genes for the GSH pathway that are absent from strain AM1. In the GSH pathway, formaldehyde is conjugated with glutathione to generate S-hydroxymethyl glutathione by Gfa (glutathione-dependent formaldehyde-activating enzyme). Then, it is converted into formate by Hgd (S-hydroxymethyl glutathione dehydrogenase) and Fgh (S-formylglutathione hydrolase), generating NADH. In addition, it has been suggested that direct oxidation of methanol to formate by XoxF takes place in *Methylacidiphilum fumariolicum* strain SolV [18], and this direct oxidation is reported to occur in an XoxF-type-specific way [33]. Another study has suggested that in strain AM1, the H_4_MPT pathway is not downregulated with La^3+^, and that in vivo formaldehyde oxidation by XoxF does not occur. However, it was also shown that ExaF can alleviate formaldehyde toxicity in an *fae* mutant strain, indicating in vivo formaldehyde oxidation [19].

Based on this background, we tried to answer the following questions: 1) Which MDH-like proteins encoded in the strain 22A genome participates in methylotrophy? 2) Is the GSH pathway functional in strain 22A as an additional route for formaldehyde oxidation? 3) Does XoxF or ExaF directly oxidize methanol into formate, and, if they do, are the formaldehyde oxidation pathways still necessary? The results of this work will increase our understanding of methylotrophy in *Methylobacterium* and related bacteria.

## 2. Materials and Methods

### 2.1. Strains and Culture Conditions

*M. aquaticum* strain 22A (FERM-BP11078) [37] was used throughout this study. Strain 22A was grown on R2A medium or mineral medium (MM) [39] containing either 0.5% methanol, 0.5% succinate, or both. *Escherichia coli* DH5α and S17-1 were grown on LB medium. Kanamycin (25 mg/L) and LaCl_3_ (30 μM) were added when necessary. For growth experiments, strain 22A and its derivatives were grown in 200 μL medium prepared in 96-well plates, which were rotary-shaken at 300 rpm at 28 °C. Growth was monitored by measuring optical density at 600 nm using a microplate reader (PowerScan HT, Sumitomo Dainippon Pharma, Osaka, Japan). MeOH-La and MeOH+La conditions refer to MM medium containing 0.5% methanol in the absence and presence of 30 μM LaCl_3_, respectively.

### 2.2. Construction of Mutant Strains

Δ*mxaF*, Δ*xoxF1*, and Δ*mxaF*Δ*xoxF1* were generated in our previous study [38]. Gene deletion mutants for *xoxF2* (Maq22A_c27990), *adh4* (Maq22A_1p32165), *exaF* (Maq22A_c07235), *adh6* (Maq22A_1p30675), and *mxbD* (Maq22A_c05310) were generated using the allele-replacement vector pK18mobSacB as previously reported [38,39]. In brief, each 1 kb upstream and downstream region of the target gene was PCR-amplified and cloned in tandem into the vector, using the primers listed in Table S1 and an In-Fusion Cloning kit (Takara Bio Co., Shiga, Japan). The vectors were introduced into strain 22A and its derivatives by conjugation using *E. coli* S17-1. Single-crossover mutants were selected by kanamycin resistance, and double-crossover mutants were selected by 10% sucrose resistance. PCR diagnosis using Up_F and Down_R primers (Table S1) was carried out as previously described [39].

We also generated re-*mxaF*, in which MxaF was left intact while the other five MDH-like genes were deleted. Similarly, re-*xoxF1*, re-*adh4*, re-*exaF*, and re-*adh6* were generated. re-0 refers to a mutant in which all six MDH-like genes were deleted. Δ*xoxF1* could grow slowly on methanol only in the presence of La^3+^, whereas re-*mxaF* could not grow irrespective of La^3+^. We isolated suppression mutants (Δ*xoxF1*Sup and re-*mxaF*Sup) from Δ*xoxF1* and re-*mxaF*, respectively; these exhibited recovered growth on methanol in the absence of La^3+^.

To determine the phenotypes of the formaldehyde oxidation-pathway mutants, a series of gene deletion mutants of formaldehyde oxidation enzyme genes, including *fae1* (Maq22A_c16490), *fae2* (Maq22A_1p31155), *mch* (Maq22A_c16475), and *hgd* (Maq22A_c21490), were generated in the wild-type (WT) and its derivatives.

### 2.3. Genome Resequencing of ΔxoxF1Sup and re-mxaFSup

The genomic DNA of Δ*xoxF1*Sup and re-*mxaF*Sup was isolated using a Wizard Genomic DNA purification kit (Promega, Tokyo, Japan) and outsourced for genome resequencing with HiSeq 2000 (paired-end, 100 bp, total ca. 20 million reads) by Macrogen Japan Co. (Kyoto, Japan). The data were mapped to six replicons of strain 22A genome sequences using BWA [40] at Maser (https://cell-innovation.nig.ac.jp). The generated BAM files were visualized with BamViewer [41]. The mutations were called with a samtools mpileup command [42].

### 2.4. Expression Vector Construction

pQF was a kind gift from Dr. J. Vorholt (Addgene plasmid #48095, ETH Zurich, Switzerland) [43]. We replaced the *tetAR* (tetracycline-resistance genes) locus of the vector with a kanamycin-resistance gene (Km) as follows. The Km was PCR-amplified with KmGeneF2 and KmGeneR2 primers using pK18mobSacB as a template. The portion of pQF except *tetAR* was amplified with pQF-KmF3 and pQF-KmR3. The resultant PCR products were combined using an In-Fusion Cloning kit to generate pQFKm. A PCR-generated *mxbD* ORF of strain 22A was introduced into the *Hin*dIII and *Bam*HI-digested vector using an In-Fusion Cloning kit. In these constructs, the N-terminal 3xFLAG sequence in the vector was eliminated, but the C-terminal sequence remained.

Meanwhile, the *tetAR* locus of the pCM130 vector (Addgene plasmid #45828, [44]) was also replaced with Km (pCM130 and pQF share the same vector backbone) to generate pCM130Km. The XylE and the T*rrnB* transcription terminator were eliminated by PCR and self-cyclization using pCM130KmC-F and pCM130Km-R primers to generate pCM130KmC, which contains an *EcoRI* site for general cloning. The *xoxF1* of strain 22A containing its promoter (ca. 1.2 kb upstream region of *xoxF1*, containing a part of *gloB* ORF) was PCR-amplified and cloned into the *Eco*RI site of the pCM130kmC. The 3′-primers contained His-Tag sequences. The resultant plasmid pCM130kmC-*xoxF1*-His was transformed into Δ*xoxF1*.

### 2.5. qPCR

Total RNA was purified from strain 22A and its derivatives using Trizol (Sigma, MO, USA). The RNA samples were treated with Promega RQ DNase I (Promega, Tokyo, Japan). cDNA synthesis was done using ReverTra Ace (Toyobo, Osaka, Japan) and a random hexamer primer. qPCR was done using the CFX Connect Real-Time PCR detection system (Bio-Rad, CA, USA), a Thunderbird SYBR green kit (Toyobo, Osaka, Japan), and primers designed for qPCR (Table S1). The thermal program was as follows: 95 °C for 1 min, followed by 45 cycles of 95 °C for 15 s and 60 °C for 30 s. A dissociation curve analysis was then conducted. The PCR-generated amplicons of each target region using the genome as a template were serially diluted and used for the generation of standard curves. Data acquisition and analysis were carried out with CFX Manager ver 3.1 (Bio-Rad, CA, USA). The expression level of each target gene was evaluated as a relative expression against *rpoC* (Maq22A_c27065)—the expression level of which was stable [38].

### 2.6. Purification of Recombinant XoxF1

Δ*xoxF1* (pCM130kmC-*xoxF1*-His) was grown in the presence of 30 μM LaCl_3_. The cells in mid- to late-log phase (OD_600_ 0.5 to 0.8) were collected by centrifugation, suspended in 50 mM potassium phosphate buffer (KPB, pH 7.0), and disrupted (4800 rpm for 30 s, 5 times) with a bead beater (Mini-BeadBeater model 3110BX, BioSpec Products, Inc., OK, USA). The homogenate was centrifuged at 20,400× *g* at 4 °C for 10 min. The supernatant was used as a cell extract and loaded onto the Ni-NTA column (3.5 mL, COSMOGEL His-accept, Nacalai Tesque, Kyoto, Japan) pre-equilibrated with buffer A (100 mM Tris-HCl pH 9.0, 150 mM NaCl, and 5 mM Imidazole). The column was washed with 20 mL buffer A and the protein was eluted with elution buffer (buffer A containing 250 mM Imidazole), 4 mL per fraction. The fractions were then desalted and concentrated with a 30 kDa-cutoff centrifugal filter tube (Amicon Ultra-15, Merck Millipore, Cork, Ireland).

### 2.7. MDH Activity and Enzyme Kinetics

The mixture, which consisted of 158 μL of 100 mM Tris-HCl pH 9.0, 2 μL of 1.5 M ammonium chloride, 10 μL of 6.6 mM phenazine methosulfate (PMS), 10 μL of 1 mM dichlorophenol indophenol (DCPIP), and 10 μL of enzyme solution (0.1 mg/mL), was plated in 96-well plates and incubated at 30 °C for 30 min, as previously described [38]. Methanol (10 μL of 20 mM) was added to start the reaction. Formaldehyde (prepared by autoclaving paraformaldehyde solution at 121 °C, 15 min) was also used as a substrate. The decrease in absorbance at 600 nm was monitored using a PowerScan HT (Sumitomo Dainippon Pharma, Osaka, Japan) microplate reader. The activity was calculated with the molar extinction coefficient for DCPIP at 600 nm of 19,000 M^−1^·cm^−1^ and then multiplied by 1.62 to convert the readings to a 1-cm light path. One unit of activity was defined as the enzyme amount that catalyzed the reduction of 1 μmol DCPIP per minute. The protein concentration was measured according to the Bradford method [45] using bovine serum albumin as the standard. The enzyme kinetics of XoxF1 were studied with varied concentrations of methanol and formaldehyde. The resultant data were fitted to non-linear regression and kinetic parameters were calculated according to the Michaelis–Menten equation.

### 2.8. S-hydroxymethyl Glutathione Dehydrogenase Assay

The reaction mixture consisted of 100 μL of 120 mM sodium phosphate (pH 7), 50 μL of 100 mM formaldehyde, and 10 μL of 100 mM glutathione [46]. The mixture was incubated at 25 °C for 10 min. NAD (5 μL of 40 mM NAD) and an appropriate amount of enzyme was added to start the reaction. The activity was measured at 340 nm (ε_NADH_ = 6220 M^−1^·cm^−1^). One unit of enzyme is defined as the amount that catalyzed the formation of 1 μmol of NADH per minute.

### 2.9. Statistical Analysis

Prism 6 (GraphPad Software, Inc., CA, USA) was used for statistical analysis.

## 3. Results

### 3.1. Phylogenetic Analysis MDH-Like Proteins of M. aquaticum Strain 22A

**Figure 2:**
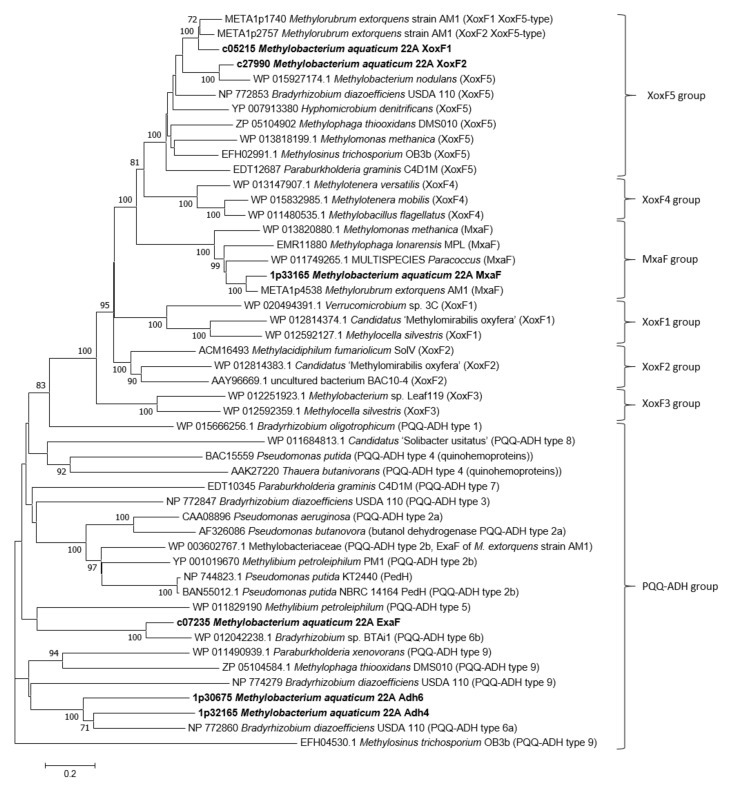
Molecular phylogenetic analysis of MDH-like proteins found in strain 22A (in boldface) and other related sequences. The evolutionary history was inferred by using the maximum likelihood method based on the Jones-Taylor-Thornton (JTT) matrix-based model [48]. The tree is drawn to scale. Evolutionary analyses were conducted in MEGA7 [49]. Bar, average number of amino acid substitutions per site.

The *M. aquaticum* strain 22A genome encodes six MDH-like genes. As previously reported in our transcriptomic and phenotypic analysis [38], *mxaF* encodes Ca^2+^-dependent MDH, *xoxF1* encodes Ln^3+^-dependent MDH, and another *xoxF1*-like gene (*xoxF2*) seems to be a pseudogene. *exaF* (Maq22A_c07235) responded to La^3+^ and seemed to be an ortholog of *exaF* found in strain AM1 [47]. PQQ-ADHs are categorized into nine types and PQQ-MDHs are classified into five subgroups (groups XoxF1–XoxF5) [33]. As shown in Figure 2, a phylogenetic analysis revealed that XoxF1 (strain 22A) is close to XoxF1 (strain AM1) and XoxF2 (strain AM1); XoxF2 (strain 22A) is close to XoxF1 of *M. nodulans*; MxaF (strain 22A) can be classified among the MxaF proteins from various methylotrophs; ExaF (strain 22A) is somewhat distant from ExaF (strain AM1, PQQ-ADH type 2b) and can be categorized as PQQ-ADH type 6b; and Adh4 (Maq22A_1p32165) and Adh6 (Maq22A_1p30675) can be categorized as PQQ-ADH type 6a. To understand which genes are involved in methylotrophy in strain 22A, we generated single-gene remaining mutants to determine their growth on methanol.

### 3.2. The Phenotype of MDH-Like Gene Mutants

**Figure 3:**
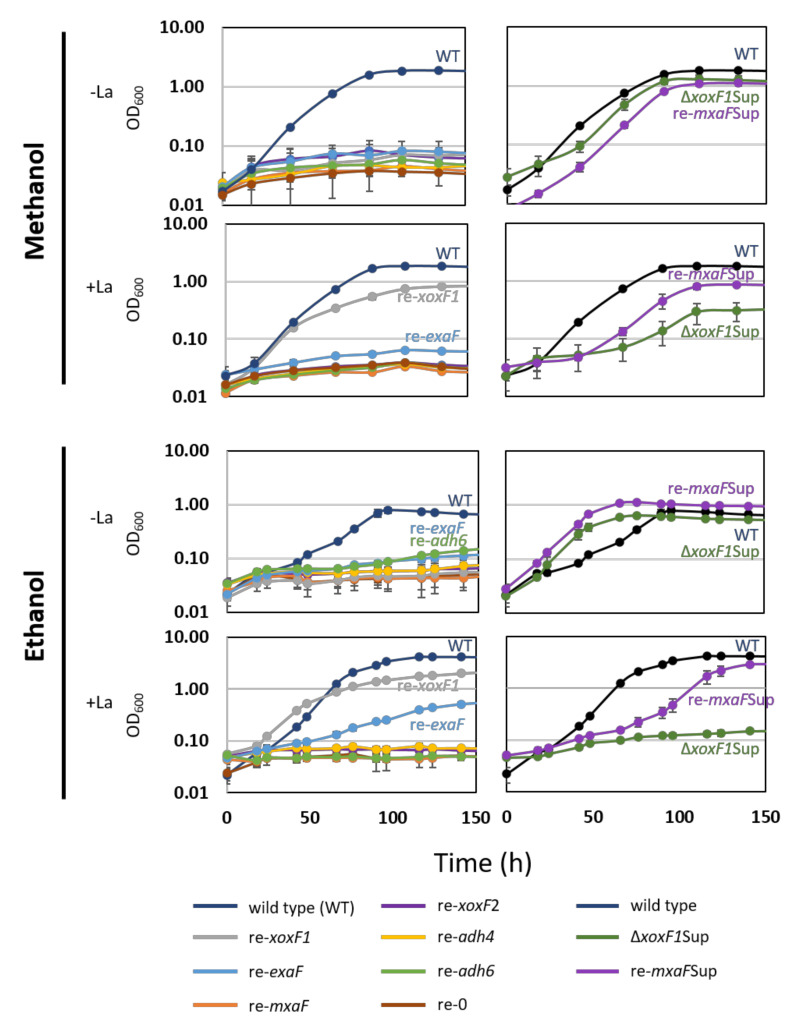
Left panel, growth of single-gene-remaining mutants of MDH-like genes and a mutant without any MDH-like gene (re-0). Right panel, growth of the suppression mutants. Cultivation was done in 96-well plates under shaking at 300 rpm. The results are presented as the average ± SD (technical triplicates).

To determine the roles of each MDH-like gene in methanol and ethanol growth, single-gene-remaining mutants of the six MDH-like genes were successfully generated, and their growth on methanol and ethanol in the presence and absence of La^3+^ was tested (Figure 3).

re-*mxaF* could not grow on methanol irrespective to the presence of La^3+^. re-*xoxF1* could grow only in the presence of La^3+^, with lower growth yield compared to the wild-type. The specific growth rate (SGR) and yield of re-*exaF* and re-0 growth on methanol were not statistically significantly different (*p* = 0.999 for both growth rate and yield, technical triplicates, data not shown), suggesting that ExaF does not contribute to methanol growth. re-*mxaF*Sup and Δ*xoxF1*Sup showed similar results, but slower growth on methanol compared to the wild-type in the absence of La^3+^. Their growth in the presence of La^3+^ resulted in slower growth and lower yield compared to the wild-type.

For ethanol growth, in the presence of La^3+^, re-*xoxF1* could grow, while re-*exaF* grew slowly with a low yield. Based on these results, we could conclude that in MM+La condition XoxF1 mainly supports the growth. The suppression mutants have an MxaF—the regulation of which is independent of the presence of *xoxF1*.

### 3.3. Mutations Detected in the Suppression Mutants

The mutation sites in re-*mxaF*Sup and Δ*xoxF1*Sup were identified through genome resequencing (Table S2). We found independent mutations in *mxbD* (Maq22A_c05310). In Δ*xoxF1*Sup, A761G caused N254S mutation. In re-*mxaF*Sup, the deletion of 12 nucleotides at positions 613–624 caused a loss of 205LLLH. These positions are located within and close to the HAMP domain (positions 215–262) of MxbD, respectively.

### 3.4. ΔmxbD Phenotype

**Figure 4:**
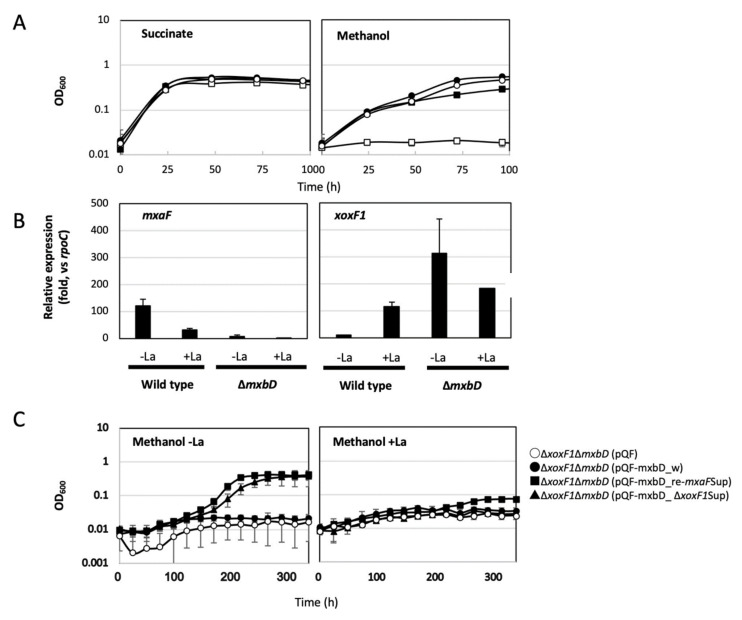
(**A**) Growth of strain 22A wild-type and Δ*mxbD* on (left panel) succinate and (right panel) methanol in the presence/absence of La^3+^. Circles, wild-type (WT); squares, Δ*mxbD*; filled symbols, in the presence of La^3+^; open symbols, in the absence of La^3+^. The results are presented as the average ± SD (technical triplicates). (**B**) Quantification of *mxaF* and *xoxF1* expression in strain 22A wild-type and Δ*mxbD*, grown on methanol in the presence/absence of La^3+^. Δ*mxbD* in +La condition was grown on methanol plus succinate. The results are presented as the average ± SD (biological triplicates). The compact letter display indicates statistically significant differences (*p* < 0.05, ANOVA and Tukey’s multiple comparison test) among the means for each gene. (**C**) Growth of Δ*xoxF1*Δ*mxbD* complemented by wild-type and mutated *mxbD* on methanol in the presence/absence of La^3+^. Open circle, vector control; closed circle, Δ*xoxF1*Δ*mxbD* (mxbD-wt); squares, Δ*xoxF1*Δ*mxbD* (*mxbD*-re-*mxaF*Sup); triangles, Δ*xoxF1*Δ*mxbD* (*mxbD*-Δ*xoxF1*Sup). The results are presented as the average ± SD (technical triplicates).

To prove that the mutations in *mxbD* were the cause of the suppression, we generated Δ*mxbD*. Δ*mxbD* showed no growth in MeOH-La conditions (Figure 4A); in MeOH+La conditions, it showed slower growth than the wild-type. The expression levels of *mxaF* and *xoxF1* in the wild-type and Δ*mxbD* were quantified by qPCR (0.5% succinate was supplemented for Δ*mxbD* in the absence of La^3+^) (Figure 4B). The wild-type showed upregulated *mxaF* and *xoxF1* expression in the absence and presence of La^3+^, respectively, showing that the Ln^3+^-switch is operating. In Δ*mxbD*, *mxaF* expression was repressed whereas *xoxF1* expression was upregulated irrespective of La^3+^. These results suggest that *mxbD* is required for *mxaF* expression and that its absence releases *xoxF1* repression.

To reproduce the suppression mutant phenotype, Δ*xoxF1*Δ*mxbD* was generated, and each *mxbD* from the wild-type (*mxbD*_w), Δ*xoxF1*Sup (*mxbD*_Δ*xoxF1*Sup), and re-*mxaF*Sup (*mxbD*_re-*mxaF*Sup) was introduced into Δ*xoxF1*Δ*mxbD*, using pQFKm as an expression vector. The mutant containing the control plasmid (pQFKm) did not grow on methanol (Figure 4C). The mutant complemented with wild-type *mxbD* did not grow as well since its genotype was the same as that of Δ*xoxF1*. The mutants complemented with the mutated *mxbD* showed growth in MeOH-La conditions. These results proved that the suppression phenotype was due to the mutations in *mxbD* and not to the other mutations detected by genome resequencing. The mutations commonly found in Δ*xoxF1*Sup and re-*mxaF*Sup (13 sites) might be due to errors of the original genome sequence, which were not confirmed in this study (Table S2).

### 3.5. The Recombinant XoxF1-His Oxidizes Methanol and Formaldehyde

To characterize the functions of XoxF1 from strain 22A, an expression vector based on pCM130KmC was designed and constructed: pCM130kmC-*xoxF1*-His was introduced into Δ*xoxF1*. Δ*xoxF1* could grow only slowly on methanol in the presence of La^3+^, but the transformant could grow well, suggesting that the introduced *xoxF1* complemented the phenotype and that the His-tagged XoxF1 is catalytically functional in vitro.

**Figure 5:**
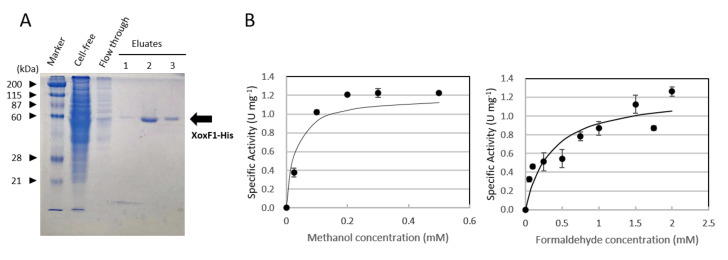
(**A**) Purification of XoxF1-His expressed in strain 22A. SDS-PAGE of crude cell extract, flow-through fraction through Ni-NTA column, and purified MDH in eluate fractions. An arrow indicates the purified XoxF1-His. (**B**) The activity of XoxF1-His against methanol and formaldehyde of varying concentrations. The data were used to calculate kinetic parameters. The results are presented as the average ± SD (technical triplicates).

The apparent monomeric molecular mass of the purified His-tagged XoxF1 (XoxF1-His) was ca. 60 kDa, as revealed by SDS-PAGE (Figure 5). The metal content of the purified XoxF1-His was determined to be 0.58 La^3+^ per monomer protein according to ICP-MS analysis. The enzyme kinetics of XoxF1-His were determined using methanol and formaldehyde as substrates. The kinetic constants (K_m_ values were 0.03 and 0.34 mM, V_max_ were 1.19 and 1.24 U·mg^−1^ for methanol and formaldehyde, respectively) indicated that XoxF1 can oxidize both methanol and formaldehyde in vitro. The kinetic parameters were within similar ranges reported for strain AM1 [19] and *Bradyrhizobium diazoefficiens* USDA110 [50].

### 3.6. hgd Encodes S-hydroxymethylglutathione Dehydrogenase

**Figure 6:**
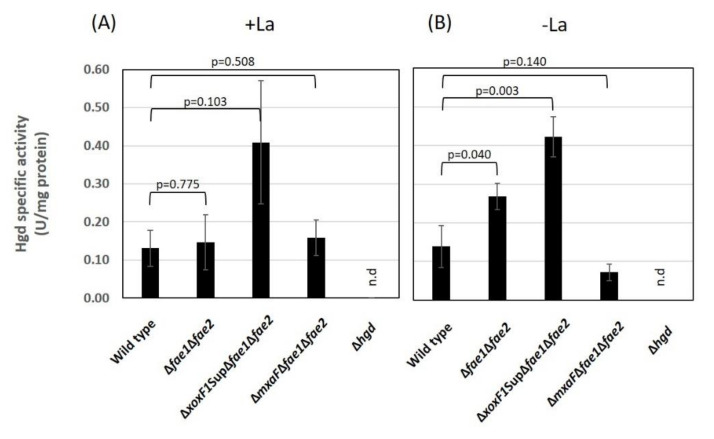
Hgd activity in the cell-free extracts of the wild-type, Δ*fae1*Δ*fae2*, Δ*xoxF1*SupΔ*fae1*Δ*fae2*, Δ*mxaF*Δ*fae1*Δ*fae2*, and Δ*hgd*, grown on methanol plus succinate in the (**A**) presence and (**B**) absence of La^3+^. Filled bar, +La^3+^; open bar, -La^3+^; n.d., not detected. The results are presented as the average ± SD (biological triplicates). Statistical tests were done with ANOVA and Tukey’s multiple comparison test independently for each dataset (presence/absence of La^3+^), and *p* values for the comparisons with the wild-type data are shown.

The GSH pathway in strain 22A is catalyzed by Gfa (Maq22A_1p37180) as the first step [51], which is expressed at a low level [38], followed by Hgd and other enzymes. As a pathway that oxidizes formaldehyde into formate, it is metabolically redundant to the H_4_MPT pathway. To determine the importance and contribution of the GSH pathway to formaldehyde oxidation, we measured the specific enzyme activity of Hgd in the cell-free extract of the cells grown on methanol plus succinate as a supplementary carbon source in the absence and presence of La^3+^ (Figure 6).

The wild-type, and Δ*fae1*Δ*fae2* in the wild-type, Δ*mxaF*, and Δ*xoxF1*Sup background were used for the assay. The wild-type showed ca. 0.1 U/mg Hgd activity in both conditions. Δ*hgd* did not show any activity, suggesting that *hgd* indeed encodes S-hydroxymethylglutathione dehydrogenase. Δ*fae1*Δ*fae2* showed increased Hgd activity in the absence of La^3+^. Interestingly, Δ*xoxF1*SupΔ*fae1*Δ*fae2* showed four times higher activity than the wild-type. However, Δ*mxaF*Δ*fae1*Δ*fae2* showed almost no change in Hgd activity. These results suggested that Hgd activity is regulated depending on formaldehyde level in the cell, which was considered to increase in Δ*fae1*Δ*fae2* mutants.

### 3.7. Characterization of the Mutants in Formaldehyde Oxidation Pathways

**Figure 7:**
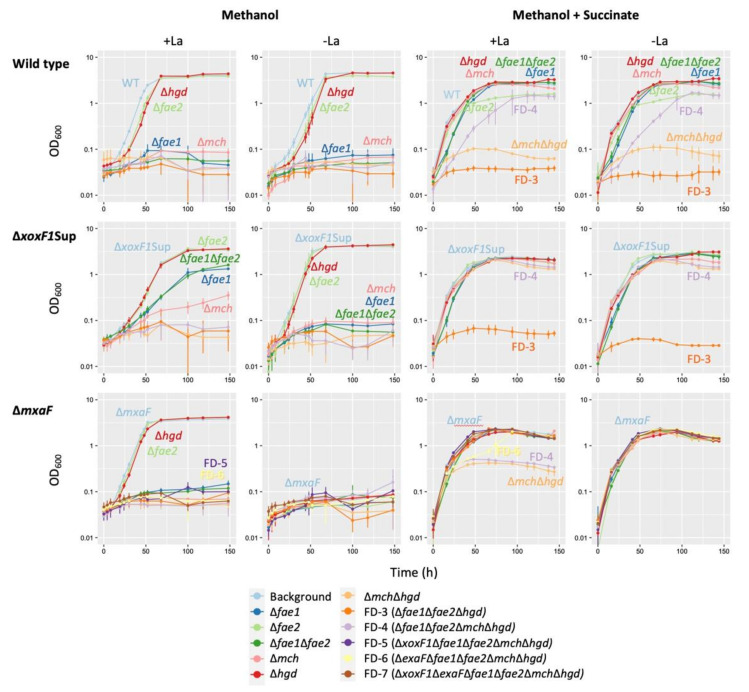
Growth of various formaldehyde oxidation-pathway mutants constructed in the wild-type, Δ*xoxF1*Sup, and Δ*mxaF* backgrounds, in methanol and methanol plus succinate liquid medium in the presence and absence of La^3+^. FD-3 (Δ*fae1*Δ*fae2*Δ*hgd*), FD-4 (Δ*fae1*Δ*fae2*Δ*mch*Δ*hgd*), FD-5 (Δ*xoxF1*Δ*fae1*Δ*fae2*Δ*mch*Δ*hgd*), FD-6 (Δ*exaF*Δ*fae1*Δ*fae2*Δ*mch*Δ*hgd*), and FD-7 (Δ*xoxF1*Δ*exaF*Δ*fae1*Δ*fae2*Δ*mch*Δ*hgd*) correspond to all three genetic backgrounds, except that FD-5 to FD-7 were constructed only for the *mxaF* background. The results are presented as the average ± SD (technical triplicates).

From the results above, we decided to use the following MDH mutants as genetic backgrounds to further study the formaldehyde oxidation pathways: Δ*xoxF1*Sup, which can grow moderately under MeOH-La conditions and slowly under MeOH+La conditions, with growth mainly dependent on *mxaF*, while its expression is de-repressed by the mutation in *mxbD;* and Δ*mxaF*, which uses mainly XoxF1 to grow on methanol only in the presence of La^3+^. We then generated single and multiple gene deletion mutants of the genes involved in formaldehyde oxidation, namely, *fae1*, *fae2*, and *mch* in the H_4_MPT pathway and *hgd* in the GSH pathway, of the wild-type, Δ*xoxF1*Sup, and Δ*mxaF*. They were grown in liquid MM containing methanol or methanol plus succinate in the presence and absence of La^3+^. The growth phenotypes and SGRs of the mutants are shown in Figure 7 and Table S3, respectively.

In the wild-type background, only Δ*hgd* and Δ*fae2* grew on methanol irrespective to La^3+^ with lower growth rates compared to the wild-type, suggesting that Hgd and Fae2 participate in the methylotrophy in the wild-type. Other mutants in the H_4_MPT pathway (*fae1* and *mch*) did not grow at all, suggesting the primary importance of the H_4_MPT pathway for the wild-type. In Δ*xoxF1*Sup background, interestingly, Δ*fae1*, Δ*fae1*Δ*fae2*, and Δ*mch* could grow slowly only in the presence of La^3+^. Because methylotrophic growth depending on ExaF was almost negligible (Figure 3), their growths were considered to be due to the de-repressed MxaF and GSH pathway. In the Δ*mxaF* background, only Δ*fae2* and Δ*hgd* showed comparable growth to the background in the presence of La^3+^. These growths were considered to be dependent mainly on XoxF1-mediated methanol oxidation and the H_4_MPT pathway. FD-5 (Δ*xoxF1*Δ*fae1*Δ*fae2*Δ*mch*Δ*hgd*) and FD-6 (Δ*exaF*Δ*fae1*Δ*fae2*Δ*mch*Δ*hgd*) could not grow on methanol as a sole carbon source. Therefore, NADH generation in formaldehyde oxidation pathways is necessary for the growth, even if XoxF1 or ExaF oxidize methanol to formate.

We then used methanol plus succinate to discriminate the reason for the growing inability of the mutants on methanol; metabolic capacity or formaldehyde toxicity. In the wild-type background, Δ*fae1*, Δ*fae2*, Δ*fae1*Δ*fae2*, Δ*mch* and Δ*hgd* could grow irrespective to La^3+^, with growth rates comparable to the wild-type. Δ*fae2* showed decreased growth yield compared to the wild-type, again suggesting its participation in methylotrophy. FD-3 (Δ*fae1*Δ*fae2*Δ*hgd*) could not grow, irrespective of La^3+^. This result suggested the involvement of the GSH pathway for formaldehyde detoxification. Interestingly, FD-4 (Δ*fae1*Δ*fae2*Δ*mch*Δ*hgd*) could grow slowly, suggesting that additional mch deletion in FD-3 alleviates formaldehyde toxicity, and possibly that methanol oxidation (formaldehyde generation) occurred slowly in FD-4. In Δ*xoxF1*Sup background, only FD-3 could not grow whereas FD-4 could grow, again implying that no formaldehyde toxicity occurs in FD-4. In the Δ*mxaF* background in the presence of La^3+^, Δ*mch*Δ*hgd* and FD-4 showed low-yield growth, suggesting that complete defect of formaldehyde oxidation pathways caused formaldehyde toxicity. This toxicity is less severe than that that occurred in Δ*mch*Δ*hgd* and FD-3 in the wild-type background and Δ*xoxF1*Sup FD-3 that were unable to grow. This difference was due to the presence of *mxaF*, suggesting that MxaF produces formaldehyde to a toxic level, and XoxF1 also does so at a lower level. FD-5 (FD-4 plus Δ*xoxF1*) did not show toxicity, whereas FD-4 did, suggesting that XoxF1 is indeed causing formaldehyde toxicity in the absence of the formaldehyde oxidation pathways. FD-6 (FD-4 plus Δ*exaF*) showed a biphasic growth, suggesting that XoxF1 causes formaldehyde toxicity to some extent but later degrades that formaldehyde. Because FD-5 grew normally, formaldehyde toxicity by ExaF did not occur. Based on the results above, XoxF1 produces formaldehyde in vivo but does not oxidize it efficiently. On the other hand, ExaF has shown to oxidize formaldehyde in vivo.

## 4. Discussion

### 4.1. Identification of PQQ-ADH Genes for Methylotrophy in Strain 22A

Through phenotyping of MDH-like gene mutants (Figure 2 and Figure 3), we concluded that strain 22A possesses a set of MDHs (MxaF and XoxF1); ExaF-type ADH that supports Ln^3+^-dependent growth on ethanol. XoxF2 (strain 22A) did not support methylotrophic growth, therefore *xoxF2* is a pseudogene or involved in other alcohol metabolism, which is different from the orthologous gene *xoxF2* in strain AM1. Methylotrophic bacteria usually possess multiple XoxF-type genes besides MxaF. XoxF5-type is commonly found in alpha-, beta-, and gammaproteobacteria [52]. XoxF4-type is found in Methylophilaceae [52]. XoxF1-, XoxF2-, and XoxF3-types are limited to specific taxa [33,53]. Additionally, strains of *Burkholderiales* contain MDH2 that oxidizes both methanol and ethanol [54]. The PQQ-ADH type 6a family, to which two proteins associated with strain 22A (Adh4 and Adh6) belong, has not been characterized. The substrate specificity of these ADHs should be investigated to reveal the ability for the utilization of different alcohols in strain 22A.

### 4.2. The Mutation in MxbD de-repress mxaF

The genomes of the suppression mutants contain mutations in the HAMP domain of MxbD (Table S2). Δ*mxbD* was unable to grow in the absence of La^3+^ and showed no expression of *mxaF* and high *xoxF1*. Therefore, MxbD is necessary for *mxaF* expression but not for *xoxF1* (Figure 4). Similar suppression mutants have been isolated from *M. extorquens* strain PA1 [30] and *P. putida* KT2440 [55]. The mutations found in the HAMP domain may confer signaling to the intracellular domain in the absence of ligand binding [56,57]. The overall regulation system for MDHs depending on the availability of substrates and metals, has not yet been clarified. It should be noted that Δ*xoxF1*Sup could be useful in further genetic studies as a mutant that has functional *mxaF* even with deleted *xoxF1*.

### 4.3. Formaldehyde Oxidation by XoxF1 and ExaF

We have confirmed that the purified recombinant XoxF1 oxidizes formaldehyde in vitro (Figure 5). In vitro activity of XoxF toward formaldehyde has also been shown in *Methylacidiphilum fumariolicum* SolV [18], *M. extorquens* AM1 [3,47], and *Bradyrhizobium diazoefficiens* USDA110 [50]. To determine whether this also occurs in vivo, we generated a series of formaldehyde oxidation pathway deficient mutants. The absence of formaldehyde toxicity in FD-3 in the Δ*mxaF* background (Figure 7) suggests XoxF1 and partly ExaF alleviate formaldehyde toxicity in vivo, implying that they oxidize formaldehyde in vivo. However, the biphasic growth of Δ*mxaF* FD-6 shows that formaldehyde oxidation by XoxF1 in vivo is not efficient. We previously showed that the GSH and H4MPT pathways in strain 22A were downregulated in the presence of La^3+^, yet cellular formaldehyde degradation activity remained high while formaldehyde production decreased [38]. These results indicated that although XoxF1 and ExaF have been shown to individually oxidize formaldehyde in vivo, ExaF may have the primary role in formaldehyde oxidation in the presence of La^3+^ when both enzymes are available. However, mutants lacking both of the formaldehyde oxidation pathways were unable to sustain their growth on methanol due to the lack of NADH generation [58].

### 4.4. The Role of the GSH Pathway

The GSH pathway for formaldehyde oxidation is considered “the most common reaction” in many organisms, including plants, mammals, and yeasts [59]. This pathway is used in methylotrophic bacteria such as *Paracoccus denitrificans* and *Rhodobacter sphaeroides* [33,51]. Some bacteria contain both GSH and H_4_MPT pathways, such as *Burkholderia phymatum* and *B. fungorum* LB400 [33,60]. In the *Methylobacterium* group, the genes for the pathway are found primarily in clade C1 (including *M. aquaticum*) and occasionally in clade A [7]. This patchy gene conservation within the genus is of interest, and its role in methylotrophy in the *Methylobacterium* group has not been investigated. We confirmed that *hgd* indeed encodes S-hydroxymethylglutathione-dehydrogenase (Figure 6). Δ*hgd* in the wild-type background showed slower growth on methanol (Figure 7). In Δ*xoxF1*Sup background, whereas Δ*fae1*Δ*fae2* could grow on methanol slowly, FD-3 could not grow, suggesting that the GSH pathway plays a role in formaldehyde oxidation. The introduction of *flhA* (GSH-and NAD-dependent formaldehyde dehydrogenase) and *fghA* (S-formyl-GSH hydrolase) from *P. denitrificans* complemented methanol growth in the H_4_MPT pathway deficient mutant of strain AM1 [11], suggesting that its role is metabolically exchangeable with that of the H_4_MPT pathway. Hgd is reported to have 1.1 U/mg activity (methanol conditions) in *P. denitrificans* [51], whereas it has ca. 0.1 U/mg activity in strain 22A. Taking these findings together, we conclude that the GSH pathway plays only a supportive role in the net formaldehyde oxidation in strain 22A. The patchy distribution of the pathway in *Methylobacterium* and related species may suggest the diminished importance of the GSH pathway in H_4_MPT pathway containing species.

### 4.5. The Role of H_4_MPT Pathway in Strain 22A

Strain AM1 *fae* mutant was unable to grow in methanol medium unless succinate was added, showing that *fae* is essential for methanol growth and that its absence causes formaldehyde toxicity [11,13], even in the presence of Ln^3+^ [19,47]. The substrate of Mch, methenyl-H_4_MPT, inhibits MtdA (NADP-dependent methylene-H_4_F dehydrogenase) in the H_4_F pathway, and thus controls the assimilation/dissimilation rate of formate [61]. Therefore, Mch is indispensable in strain AM1.

In strain 22A, Fae1 and Mch are also found to be essential for the wild-type to grow on methanol. However, Δ*fae1*, Δ*fae1*Δ*fae2*, and Δ*mch* in the Δ*xoxF1*Sup background could grow on methanol in the presence of La^3+^. We concluded that the H_4_MPT pathway is not essential when the methylotrophic growth is dependent only on de-repressed MxaF in strain 22A with a GSH pathway in the presence of La^3+^.

### 4.6. Overall Methanol Metabolism in Strain 22A

Through this study, we first revealed that among the six MDH-like proteins in strain 22A, two MDHs (MxaF and XoxF1) are involved in methanol oxidation. Second, MxbD is necessary for *mxaF* expression and *xoxF1* repression, and a suppression mutant Δ*xoxF1*Sup enabled us to use it as a MxaF-expressing mutant without *xoxF1*. Third, the GSH pathway is functional and plays a role in formaldehyde oxidation and the generation of NADH; due to the low expression, however, its role is supportive for the net formaldehyde oxidation in addition to the H_4_MPT pathway that holds primary importance. Fourth, XoxF1 is capable of formaldehyde oxidation in vivo and in vitro and alleviates formaldehyde toxicity in formaldehyde oxidation-pathway mutants but in the absence of the NADH-producing pathways, it cannot solely support methanol growth. ExaF is involved in Ln^3+^-dependent ethanol growth and formaldehyde detoxification. In a natural environment, these redundant oxidation pathways catalyzed by different MDHs and formaldehyde oxidation pathways would enable cells to control the carbon flux in the redundant pathways, depending on the availability of Ln^3+^, the intracellular level of formaldehyde, and the required NADH level.

## Supplementary Materials

The following are available online at https://www.mdpi.com/2076-2607/8/6/822/s1: Table S1, Primers used in this study; Table S2, Mutations detected in the suppression mutants by genome resequencing; Table S3, Specific growth rates (SGRs) and cell yield of various formaldehyde oxidation mutants grown on methanol and methanol plus succinate in the presence and absence of La^3+^.
